# Synthesis and Characterization of the Conducting Polymer Micro-Helix Based on the *Spirulina* Template

**DOI:** 10.3390/polym10080882

**Published:** 2018-08-07

**Authors:** Xiao-Yu Hu, Jun Ouyang, Guo-Chang Liu, Meng-Juan Gao, Lai-Bo Song, Jianfeng Zang, Wei Chen

**Affiliations:** 1 College of Life Science and Technology, Huazhong University of Science and Technology, Wuhan 430074, China; 13971144169@139.com (X.-Y.H.); m201571649@hust.edu.cn (J.O.); d201577407@hust.edu.cn (G.-C.L.); m201671630@hust.edu.cn (M.-J.G.); m201771732@hust.edu.cn (L.-B.S.); 2 Hubei Boffin Technology Co. Ltd., Wuhan 430074, China; 3 Innovation Institute, Huazhong University of Science and Technology, Wuhan 430074, China; jfzang@hust.edu.cn

**Keywords:** PPy micro-helix, PANI micro-helix, PEDOT micro-helix, *Spirulina*, bio-template, conducting polymer

## Abstract

As one of the most interesting naturally-occurring geometries, micro-helical structures have attracted attention due to their potential applications in fabricating biomedical and microelectronic devices. Conventional processing techniques for manufacturing micro-helices are likely to be limited in cost and mass-productivity, while *Spirulina*, which shows natural fine micro-helical forms, can be easily mass-reproduced at an extremely low cost. Furthermore, considering the extensive utility of conducting polymers, it is intriguing to synthesize conducting polymer micro-helices. In this study, PPy (polypyrrole), PANI (polyaniline), and PEDOT (poly(3,4-ethylenedioxythiophene)) micro-helices were fabricated using *Spirulina* *platensis* as a bio-template. The successful formations of the conducting polymer micro-helix were confirmed using scanning electron microscopy (SEM). Fourier transform infrared spectroscopy (FTIR) and Raman and X-ray diffraction (XRD) were employed to characterize the molecular structures of the conducting polymer in micro-helical forms. In the electrochemical characterization, the optimized specific capacitances for the PPy micro-helix, the PANI micro-helix, and the PEDOT micro-helix were found to be 234 F/g, 238 F/g at the scan rate of 5 mV/s, and 106.4 F/g at the scan rate of 10 mV/s, respectively. Therefore, it could be expected that other conducting polymer micro-helices with *Spirulina* as a bio-template could be also easily synthesized for various applications.

## 1. Introduction

Due to their underlying chemical and physical functions, the helical structure is one of the most interesting geometries discovered in many biomolecules and bio-structures, such as the α-helix structure in protein, dsDNA, virus, bacteria, spiral vessels, tendrils, etc. [1]. Inspired by these naturally-occurring structures, considerable efforts have been made to design and fabricate various microscale or nanoscale helix-based devices, such as micromotors [2,3,4,5,6,7,8], circular polarizers [9], force sensors [10,11,12], actuators [13,14], and artificial muscles [15,16]. Furthermore, the theoretical calculations on helix-based materials have also predicted other possible applications including catalysis [17], plasmonics [18], circular dichroism [19], and reflection [20].

In order to generate helical micro/nano-structures, various techniques, including molecular self-assembly, electrospinning [21], photolithography [22], mechanical twisting [23], microfluidics [1,24], and oblique angle-based deposition [25] have been developed. However, it is still difficult to realize the large-scale production of a uniform helix at a low cost since most of these techniques are based on delicate conditions and sophisticated equipment [26]. Thus, the high requirements for these fabrication approaches are expected to limit their practical applications. On the one hand, there are plenty of highly-ordered biological structures in a wide range of dimensions and patterns that can be employed as templates [27]. For micro-sized helix synthesis, two kinds of biological templates have mainly been used: one is from xylem vessels that are composed of chiral crystalline cellulose and widely distributed in almost every part of vascular plants [28], and the other is from *Spirulina* that grows naturally in a helical shape and has already been commercialized as food supplements [29]. Though xylem-originated helical microstructures are abundant in a wide variety of plants, the difficulty in the isolation process of the spiral vessels through chemical methods still exist [28]. In contrast, *Spirulina* that is in a perfect helical shape can be easily cultured and extracted in large quantities by simple filtration. Until now, *Spirulina*-based templates have successfully contributed to the synthesis of the magnetic helix [4,30], silica helix [31], copper helix [32,33,34], Ni-Fe-P alloy helix [35], and silver helix [36].

On the other hand, conducting polymers, such as polypyrrole (PPy), polyaniline (PANI), poly(3,4-ethylenedioxythiophene) (PEDOT), polythiophene (PTh), and poly(*p*-phenylenevinylene) (PPV) have been widely fabricated or integrated into micro/nanostructure-based devices for the applications of flexible supercapacitors [37,38], biofuel cells [39], artificial muscles [40], tissue scaffolds [41], artificial synapses [42], smart textiles [43], etc. The conducting polymers in helical micro/nanostructures have also been reported using both template- and non-template-based methods [21,44,45]. Among these methods, the templates are critical for the fabrication of highly-ordered helical microstructures of the conducting polymers [45,46,47,48]. Thus, it will be interesting and worthy to utilize *Spirulina* as a helical bio-templates which can be easily reproduced *en masse* and with low cost.

In this article, *Spirulina*-templated conducting polymer micro-helices, including PPy micro-helix, PANI micro-helix, and PEDOT micro-helix, were successfully synthesized by in situ polymerization using FeCl_3_ as an oxidant. The morphologies, molecular structures, and electrical and electrochemical properties of the conducting polymer micro-helices were characterized by SEM, FTIR, Raman, XRD, four-point probes, CV, and EIS, respectively.

## 2. Materials and Methods

### 2.1. Materials

**Figure 1:**
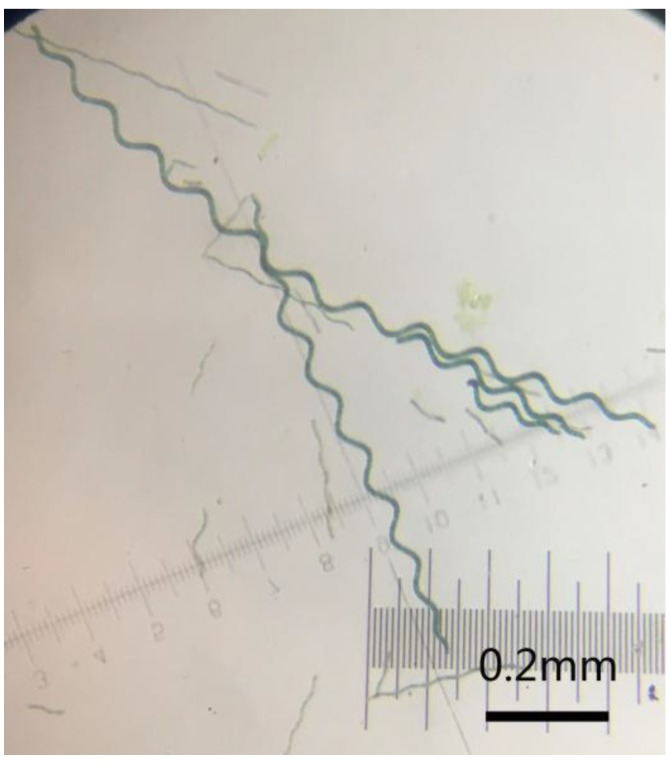
The optical microscope images of *Spirulina* cells.

*Spirulina platensis* (FACHB-439) was purchased from the Institute of Hydrobiology, Chinese Academy of Sciences (Wuhan, China). Figure 1 shows the optical microscope image of *Spirulina platensis* in its natural form and its wire diameter, diameter of helix, pitch, turn number, free length, and pitch angle are observed to be about 5–7, 20–25, 24–30, 8–16, 176–780 μm and 90°–120°, respectively. A modified Zarrouk medium for *Spirulina* cultivation contained 13.61 g/L sodium bicarbonate (NaHCO_3_), 4.03 g/L sodium carbonate (Na_2_CO_3_), 0.50 g/L dipotassium hydrogenphosphate (K_2_HPO_4_), 2.50 g/L sodium nitrate (NaNO_3_), 1.00 g/L potassium sulfate (K_2_SO_4_), 1.00 g/L sodium chloride (NaCl), 0.20 g/L magnesium sulfate heptahydrate (MgSO_4_·7H_2_O), 0.04 g/L calcium chloride dehydrate (CaCl_2_·2H_2_O), 0.01 g/L ferrous sulfate heptahydrate (FeSO_4_·7H_2_O), 2.86 μg/L orthoboric acid (H_3_BO_3_), 1.86 μg/L manganese chloride tetrahydrate (MnCl_2_·4H_2_O), 0.22 μg/L zinc sulphate heptahydrate (ZnSO_4_·7H_2_O), 0.39 μg/L sodiummolybdate dehydrate (Na_2_MoO_4_·2H_2_O), 0.08 μg/L copper(II) sulfate pentahydrate (CuSO_4_·5H_2_O), and 0.05 μg/L cobaltous nitrate hexahydrate (Co.(NO_3_)_2_·6H_2_O). Additionally, all these reagents were bought from Sinopharm Chemical Reagent Co., Ltd. (Shanghai, China). Pyrrole, aniline, and 3,4-ethylenedioxythiophene were purchased from the Aladdin Industrial Corporation (Shanghai, China). Polytetrafluoroethylene with an average particle size of 5 μm was purchased from Shanghai Macklin Biochemical Co. Ltd. (Shanghai, China). Nickel foam (99.8% Ni) and acetylene black (battery grade) were purchased from the Taiyuan Liyuan Lithium Technology Center Co. Ltd. (Taiyuan, China). Deionized water (18.2 MΩ·cm, Milli-Q Reference, Merck Millipore, Shanghai, China) was used throughout the experiments.

### 2.2. Methods

#### 2.2.1. Cultivation and Preparations of *Spirulina platensis*

The cultivation of the *Spirulina* started with the initial inoculum density of 0.1 with magnetic stirring in an illumination incubator (Ningbo Yanghui instrument Co., Ltd., Ningbo, China) at 36 ± 1 °C in 12/12 h light-dark cycles. When the inoculum density was above 1.2 after 12 days of cultivation, the *Spirulina* was collected by a 350-mesh nylon filter and rinsed with 0.9% *w*/*w* sodium chloride solution three times. The tissue fixation of the *Spirulina* was carried out in 1:9 *v*/*v* glutaraldehyde aqueous solution for 6 h to maintain the bio-helix form. After the fixation, the unreacted glutaraldehyde was rinsed out with 0.01 M phosphate-buffered solution (PBS) and deionized water, successively. Fixed *Spirulina* template suspension was used throughout the experiments.

#### 2.2.2. Fabrication of the *Spirulina*-Templated PPy Helical Microstructure

For the fabrication of *Spirulina*-templated PPy helical microstructure, 11 g of *Spirulina* template was suspended in a 50 mL solution containing 0.1 M HCl and 0.2 M pyrrole by magnetic stirring at 300 rpm for 0.5 h. For PPy polymerization, 50 mL of 0.6 M FeCl_3_ solution was slowly added into the suspension and reacted for 6 h. After the polymerization, the suspension was filtered by a 350-mesh nylon filter and rinsed with 0.1 M HCl to remove the loosely bound or uncoated PPy. Finally, the PPy micro-helix was dried in an oven at 50 °C for 6 h.

#### 2.2.3. Fabrication of the *Spirulina*-Templated PANI Helical Microstructure

For the fabrication of *Spirulina*-templated PANI helical microstructure, 12 g of *Spirulina* template was suspended in 160 mL of water containing 0.5 M aniline by magnetic stirring at 300 rpm for 1 h. The aniline adsorbed *Spirulina* cells were added into 150 mL of 0.5 M FeCl_3_ solution and reacting overnight with magnetic stirring. After the polymerization, the suspension was filtered by a 350-mesh nylon filter and sonicated in ethanol to dislodge loosely bound or free polymers. The suspension was then washed with 0.1 M HCl and deionized water, successively. Finally, the PANI micro-helix was dried in an oven at 50 °C for 6 h.

#### 2.2.4. Fabrication of the *Spirulina*-Templated PEDOT Helical Microstructure

For the fabrication of *Spirulina*-templated PEDOT helical microstructure, 30 mL of a suspension containing 2.5 g of *Spirulina* template, 0.23 M 3,4-ethylenedioxythiophene (EDOT), 2.34 M FeCl_3_·6H_2_O, and 0.12 M *p*-toluenesulfonic acid monohydrate were mixed. For the PEDOT polymerization, the suspension was sonicated in an ultrasonic washer for 1 h. After the polymerization, the suspension was filtered by a 350-mesh nylon filter and washed with deionized water and pure ethanol successively to remove the loosely bound or free polymers. Finally, the PEDOT micro-helix was dried in an oven at 50 °C for 6 h.

#### 2.2.5. Preparations of PPy, PANI, and PEDOT Helix Modified Electrodes

The PPy, PANI, and PEDOT micro-helix powders, acetylene black, and poly(tetrafluoroethylene) (PTFE) were mixed by the mass ratio of 8:1:1, and ground homogeneously with the addition of pure ethanol, respectively. For each conducting polymer micro-helix, about 0.01 g of the mixture was coated onto a nickel foam sheet in the size of 1 cm × 1 cm and oven-dried at 60 °C overnight.

### 2.3. Characterizations

#### 2.3.1. Morphology Analysis

The morphology of *Spirulina platensis* in its natural form was observed using an optical microscope (SMARTR, Chongqing Ott optical instruments Co., Ltd., Chongqing, China). The morphologies of the PPy micro-helix, PANI micro-helix, and PEDOT micro-helix were observed using a field emission scanning electron microscope (FESEM) (Helios NanoLab G3, FEI Company, Brno, Czech Republic and Nova Nano SEM 450, FEI Company, Eindhoven, The Netherlands) with the accelerating voltages of 2 and 10 kV.

#### 2.3.2. Fourier Transform Infrared Spectroscopy (FTIR)

A Fourier transform infrared spectrometer (Vertex 70, Bruker Corporation, Karlsruhe, Germany) was used to obtain the FTIR spectra of the PPy, PANI, and PEDOT micro-helices with a scanning range of 400–4000 cm^−1^ at a resolution of 1.93 cm^−1^ at room temperature. The sample tablets were prepared by mixing the sample powders with 5% (*w*/*w*) potassium bromide (KBr).

#### 2.3.3. Raman Spectroscopy

A confocal laser Raman spectrometer (LabRAM HR800, Horiba Jobin Yvon, Paris, France and Renishaw inVia plus, Renishaw plc, Gloucestershire, United Kingdom) was employed to obtain a Raman Spectroscopy of the PPy, PANI, and PEDOT micro-helices. Raman spectra were acquired with a 532 nm excitation laser source with a power of 1.8 mW in the range of 500–2000 cm^−1^.

#### 2.3.4. X-ray Diffraction (XRD)

An X-ray diffraction meter (Empyrean XRD, Malvern Panalytical Ltd., Malvern, UK) was used to determine the atomic and molecular structure of the PPy, PANI, and PEDOT micro-helices. The XRDs were conducted with a Cu *K*-Alpha radiation source with a step size of 0.01313° in the scanning range of 5°–50° and wavelength of 1.540598 Å.

#### 2.3.5. Electrochemical Characterization

The sheet resistance of each conducting polymer micro-helix was measured in three different areas, three times by four-point probes (ST2263 digital four-point probes, Suzhou Jingge Electronic Co., Ltd., Suzhou, China). Cyclic voltammetry (CV) and electrochemical impedance spectroscopy (EIS) of the PPy, PANI, and PEDOT micro-helix-modified electrodes were conducted on a CS 1350 electrochemical workstation (Wuhan Corrtest Instrument Co., Ltd., Wuhan, China) with a three-electrode cell in which a Pt foil (1 cm × 1 cm) and Ag/AgCl electrode were used as a counter electrode and reference electrode, respectively. In CV measurements, ten cycles between −0.3 and 0.7 V in 0.1 M Na_2_SO_4_ at scan rates of 5, 10, 20, 30, 50, and 100 mV/s were performed. EIS measurements were performed in the frequency range of 10^5^ to 0.01 Hz at an open circuit potential with an AC perturbation of 10 mV, and the data were analyzed by ZView software (Wuhan Corrtest Instrument Co., Ltd., Wuhan, China).

## 3. Results and Discussion

### 3.1. Morphologies of PPy, PANI, and PEDOT Micro-Helices

**Figure 2:**
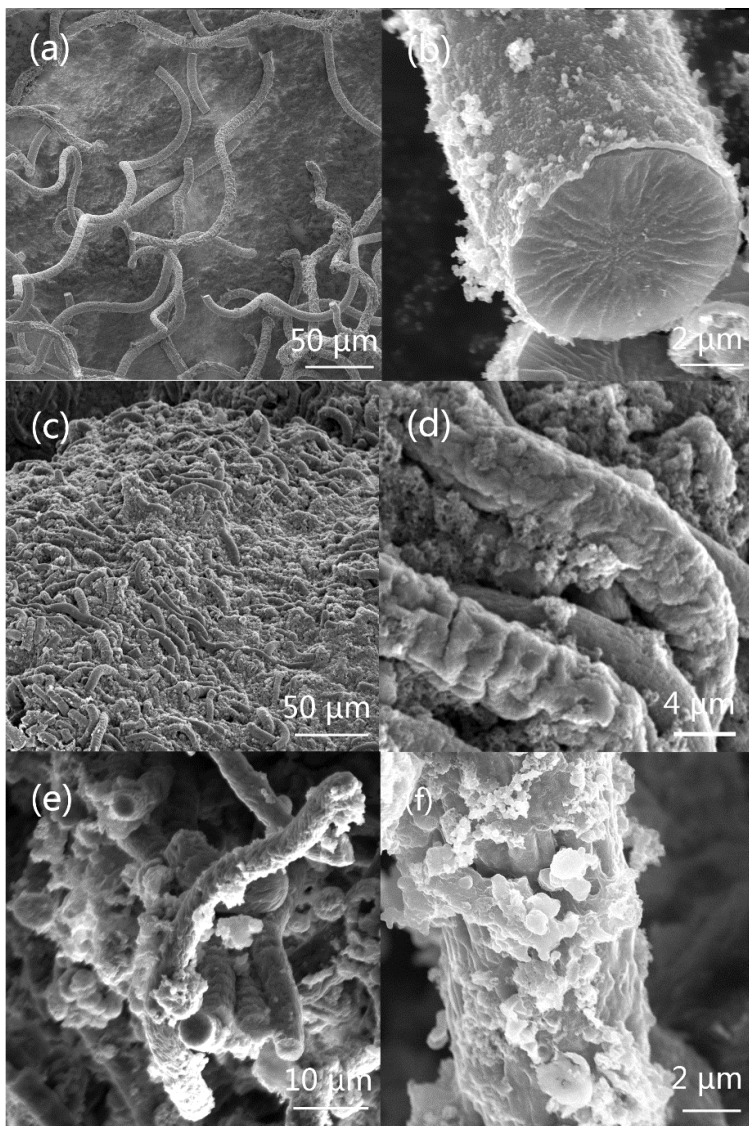
The scanning electron microscopy (SEM) images of polypyrrole (PPy) micro-helix (**a**,**b**); polyaniline (PANI) micro-helix (**c**,**d**); and poly(3,4-ethylenedioxythiophene) (PEDOT) micro-helix (**e**,**f**).

In Figure 2a, the distinct display of the helix micro-structures without any metal sputtering coatings confirmed that conductive PPy has been successfully formed on the surface of *Spirulina* and the coating process showed no adverse effect on the initial helical shape of *Spirulina*. In Figure 2b, it can be observed that the cross-section of the PPy micro-helix has a solid and compact interior which has a diameter of about 7.3 µm and a relatively thin outer layer with about 0.3 µm thickness. The appearance of this solid interior is interesting because both the hollow and the solid helical structures have been observed on Cu-coated *Spirulina* and Ag-coated *Spirulina* by electroless plating [32,34,36]. This solid interior of the helical structure should be composed of the fixed cytoplasmic components from glutaraldehyde treatment because it also existed in the *Spirulina* templates without PPy coating. This difference between the hollow and solid interiors may result from the *Spirulina* template preparation process. In the hollow helical structure formation, the drying process vaporized the moisture of *Spirulina* and the volume of *Spirulina* shrunk to form the hollow helical structure [34], while in our preparation, no drying process was included. PPy coating shows a typical granular raspberry morphology and a fully-encapsulated *Spirulina* surface [49]. As no bare *Spirulina* can be observed and the PPy layers fully covered on all these helical template surfaces, it can be speculated that there exists a chemical bonding between PPy and the *Spirulina* surface. As one genre of cyanobacteria, *Spirulina* is also covered by the external surface layers that are composed of 2D crystalline arrays of glycoprotein (S-layers) and carbohydrate structures [50,51]. Though the compositions of these carbohydrate structures may vary with the species of cyanobacteria, they mainly include monosaccharides and polysaccharides, such as cellulose-like homoglucan, xyloglucan, uronic acid, glucose, xylose, and ribose [52]. Therefore, this bonding is likely to be hydrogen bonding that is from the interaction between the hydroxyl groups of the saccharides in the *Spirulina* cell wall and the H of the N and/or the N in the pyrrole ring [53]. Figure 2c shows that the *Spirulina* templates were not only enwrapped, but also bounded with each other by PANI. Though aniline monomers are likely to initiate the polymerization on the hydroxyl groups of the saccharides on the *Spirulina* surface, the free PANI seems to be easily formed between the spaces of the *Spirulina* templates, as shown in Figure 2d. This result may be due to the relatively high aniline monomer concentration since the morphology of the PANI-coated cotton fiber was also monomer concentration-dependent [54]. In Figure 2e, it can be seen that the *Spirulina* templates were coated with an uneven granular PEDOT and their cross-sections also showed solid and compact interiors since they had the same preparation process as that of the PPy micro-helix. In Figure 2f, a rougher surface of PEDOT micro-helix can be found in comparison with the PPy and PANI micro-helix surfaces. This morphology may suggest that there exists the weakest interaction between the PEDOT and *Spirulina* surfaces among these three conducting polymers.

### 3.2. Fourier Transform Infrared Spectroscopy (FTIR) Analysis

**Figure 3:**
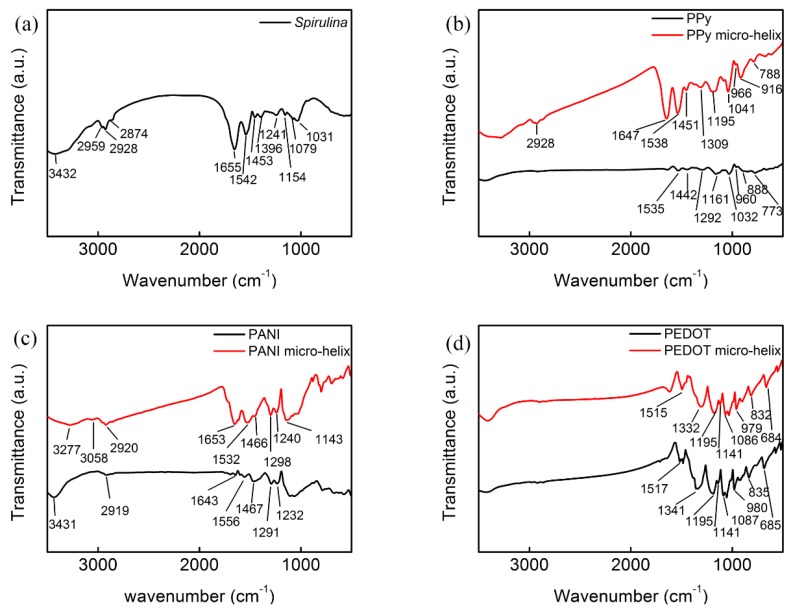
The Fourier transform infrared spectroscopy (FTIR) spectrum of *Spirulina* (**a**); PPy and the PPy micro-helix (**b**); PANI and the PANI micro-helix (**c**); and PEDOT and the PEDOT micro-helix (**d**).

In Figure 3a, the broad band at around 3432 cm^−1^ of raw *Spirulina* was assigned to –OH groups of glucose and –NH groups of protein [55,56]. The bands at 2959, 2928, and 2874 cm^−1^ correspond to lipid methyl (–CH_3_) asymmetric stretching, lipid methylene (–CH_2_) asymmetric stretching, and protein methyl (–CH_3_) symmetric stretching, respectively [57]. The bands at 1655, 1542, and 1453 cm^−1^ can be assigned to C=O stretching, and N-H inner and outer bending, respectively [55,58]. The bands at 1396, 1241, 1154, 1079, and 1031 cm^−1^ can be attributed to C–O, C–C stretching vibrations of protein, fatty acids, and saccharides, respectively [57,59].

FTIR spectra of free PPy and PPy micro-helix are shown in Figure 3b. A broad vibrational band between 3200–3500 cm^−1^ can be assigned to N–H stretching vibrations [56]. The bands in the PPy micro-helix at about 2928 and 1647 cm^−1^ can be assigned to *sp*^3^ C–H stretching and N–H bending vibration, respectively [60]. Free PPy exhibits a distinct band at 1535 cm^−1^, which is attributed to C–C stretching vibrations in the pyrrole ring, while a small blue shift to 1538 cm^−1^ of this band is observed in the PPy micro-helix [61]. The bands at 1442 and 1451 cm^−1^ in the spectra of PPy and the PPy micro-helix correspond to C–N stretching vibrations in the ring [61]. PPy and the PPy micro-helix have broad bands in the range of 1400–1250 cm^−1^ which can be assigned to C–H or C–N in-plane deformation modes with the maximum at 1292 and 1309 cm^−1^, respectively [62]. The bands between 1250 to 1100 cm^−1^ correspond to the breathing vibration of the pyrrole ring with the maximum at 1161 and 1195 cm^−1^ for PPy and the PPy micro-helix, respectively [62]. The bands of aromatic C–H and N–H in the plane deformation vibration situate in the region from 1130 to 1000 cm^−1^ for the PPy micro-helix and PPy [62,63]. The bands at 966 and 960 cm^−1^ can be assigned to C–C out-of-plane ring deformation vibrations of the PPy micro-helix and PPy, respectively [63]. The bands of C–H out-of-plane deformation vibrations have maximums at 916 cm^−1^ and 888 cm^−1^ for the PPy micro-helix and PPy, respectively. The peaks at 788 and 773 cm^−1^ correspond to the C–H out-of-plane ring deformation of the PPy micro-helix and PPy, respectively [63].

FTIR spectra of free PANI and the PANI micro-helix are shown in Figure 3c. The peak at 3431 cm^−1^ for free PANI can be assigned to N–H stretching vibration [64]. The bands at 3277 and 3058 cm^−1^ in the spectrum of the PANI micro-helix are due to hydrogen-bonded N–H stretching and aromatic C–H stretching, respectively [65]. Free PANI exhibits two distinct bands at 2919 and 1643 cm^−1^ which are attributed to the asymmetrically stretching vibration of C–H and the O–H bending of the absorbed water, respectively [66], while these bands show a slight blue-shift to 2920 and 1652 cm^−1^ in the spectrum of the PANI micro-helix. The absorption bands at 1556 and 1467 cm^−1^ in the spectrum of PANI can be assigned to the stretching vibrations of the quinoid (N=Q=N) and benzoid (N–B–N) structure, respectively, while these bands have redshifts to 1532 and 1466 cm^−1^ in the PANI micro-helix, respectively. This result may suggest the existence of π stacking interaction between PANI and *Spirulina* [67]. The band at 1298 cm^−1^ in the spectrum of the PANI micro-helix corresponds to π-electron delocalization which is caused by polymer protonation [63]. The band at 1291 cm^−1^ can be assigned to the bending vibrations of the N–H mode of the polaron structure on the PANI micro-helix [68]. The band at 1240 cm^−1^ corresponds to the asymmetric C–N^+^ stretching vibration in the polaron structure on the PANI micro-helix and slightly shifts to 1232 cm^−1^ in the spectrum of PANI [63,69]. A specific band at 1143 cm^−1^ observed in the PANI micro-helix can be attributed to a vibration mode of the N–H structure which is absent in PANI [69].

FTIR spectra of free PEDOT and the PEDOT micro-helix are shown in Figure 3d. The bands at 1517 and 1341 cm^−1^ are attributed to the C=C stretching modes or C–C stretching of the quinoid structure in the thiophene ring, while the bands shift to 1515 and 1332 cm^−1^ in the spectrum of PEDOT micro-helix [70,71]. Peaks at 1195, 1141, and 1087 cm^−1^ that are due to C–O–C bond stretching in the ethylene dioxy group exist in both PEDOT and the PEDOT micro-helix [71]. The bands at 980, 835, and 685 cm^−1^ in the spectrum of PEDOT can be attributed to the deformation modes of the C-S bond in the thiophene ring which shift to 979, 832, and 684 cm^−1^ in the spectrum of the PEDOT micro-helix, respectively [70]. The bands around 3400 cm^−1^ in both PEDOT and the PEDOT micro-helix are attributed to the vibration of H_2_O and suggest water residue in the samples [72]. In addition, the band at 1640 cm^−1^ is ascribed to the C=C stretching vibration which is strongly affected by the doping level of the conducting polymer [73] and suggests a higher doping level of the PEDOT micro-helix in comparison with that of PEDOT.

### 3.3. Raman Spectroscopy Analysis

**Figure 4:**
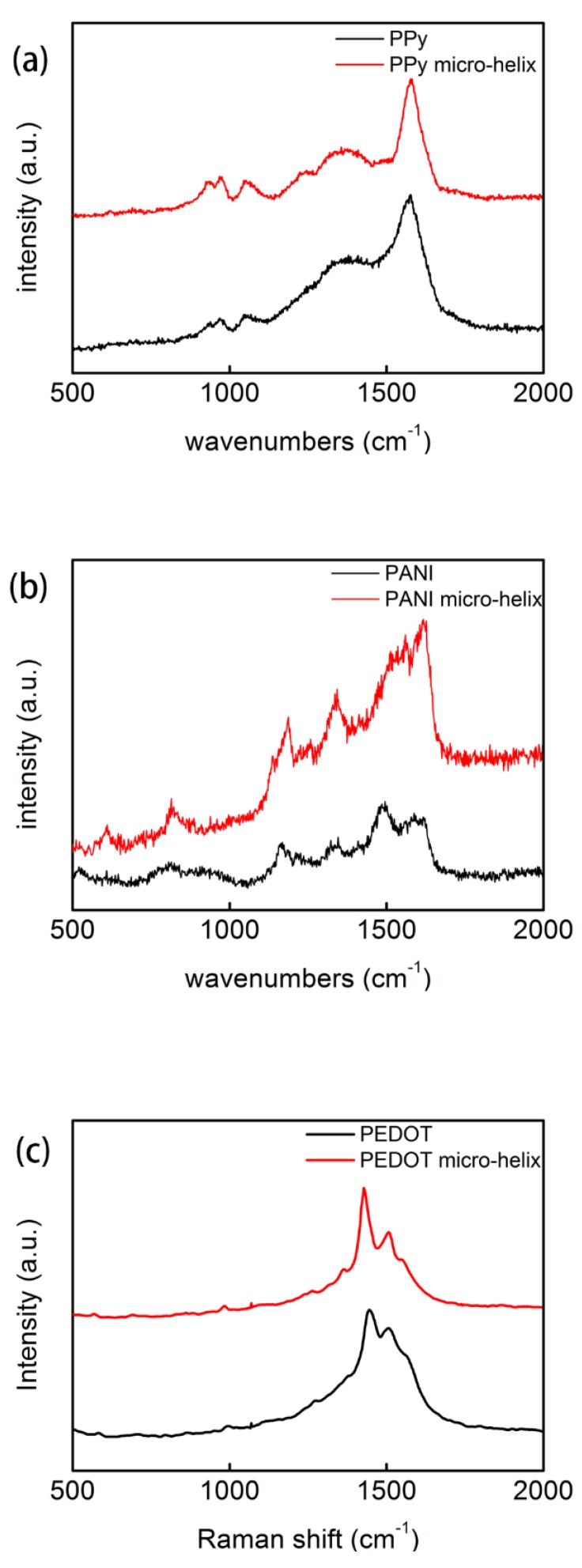
The Raman spectra of PPy and the PPy micro-helix (**a**); PANI and the PANI micro-helix (**b**); and PEDOT and the PEDOT micro-helix (**c**).

In Figure 4a, the band at 1577 cm^−1^ is assigned to the C=C backbone stretching of PPy, and the band at 1380 cm^−1^ is attributed to the ring stretching mode for both PPy and the PPy micro-helix [74]. The band at 1240 cm^−1^ in the PPy micro-helix can be assigned to the anti-symmetrical C–H in-plane bending [75]. In both PPy and the PPy micro-helix, the peaks at 1050 cm^−1^ represent the symmetrical C–H in-plane deformation and the peaks at 968 cm^−1^ are associated with the polaron structure [74,75]. The peak at 938 cm^−1^ in PPy and the peak at 935 cm^−1^ in the PPy micro-helix are attributed to ring deformation induced by dication (dipolaron) [76].

In Figure 4b, the bands at 1617 cm^−1^ in both PANI and the PANI micro-helix spectra are attributed to C–C stretching [77]. The bands at 1588 cm^−1^ in PANI and at 1513 cm^−1^ in the PANI micro-helix may be associated with C=C stretching in the quinonoid ring [77,78], and the bands at 1562 cm^−1^ in PANI and at 1560 cm^−1^ in the PANI micro-helix can be attributed to the C–C stretching of the intermediate structure between quinoid and semiquinoid [79]. The band at 1494 cm^−1^ in PANI represents benzenoid ring vibration (C=C stretching deformations) [80]. The bands at 1345 cm^−1^ in PANI and 1342 cm^−1^ in the PANI micro-helix can be attributed to C–N^+^ stretching of radical cations [81]. The bands at 1187 cm^−1^ in the PANI micro-helix and at 1166 cm^−1^ in PANI can be assigned to C–H in-plane bending vibrations of bipolaronic forms and C–H bending quinoid, respectively [81,82]. The bands at 814, 610, and 520 cm^−1^ in both the spectra may result from the C–N–C bending, ring deformation, and amine in-plane deformation, respectively [82,83].

In Figure 4c, the band of C=C symmetrical stretching in the thiophene ring is at 1444 cm^−1^ in PEDOT and shifts to 1426 cm^−1^ in the PEDOT micro-helix [84,85]. The bands of asymmetrical C=C stretching are at 1506 cm^−1^ for both PEDOT and the PEDOT micro-helix [86,87]. The peak at 1361 cm^−1^ that is related to C–C stretching is only observed in the PEDOT micro-helix [87]. The peaks at 1265 cm^−1^ in the PEDOT micro-helix and 1274 cm^−1^ in PEDOT are assigned to the C–C inter-ring stretching mode [85]. The peaks of the C–C anti-symmetrical stretching mode can be seen at 983 cm^−1^ in the PEDOT micro-helix and 994 cm ^−1^ in PEDOT [85].

### 3.4. X-ray Diffraction Analysis

**Figure 5:**
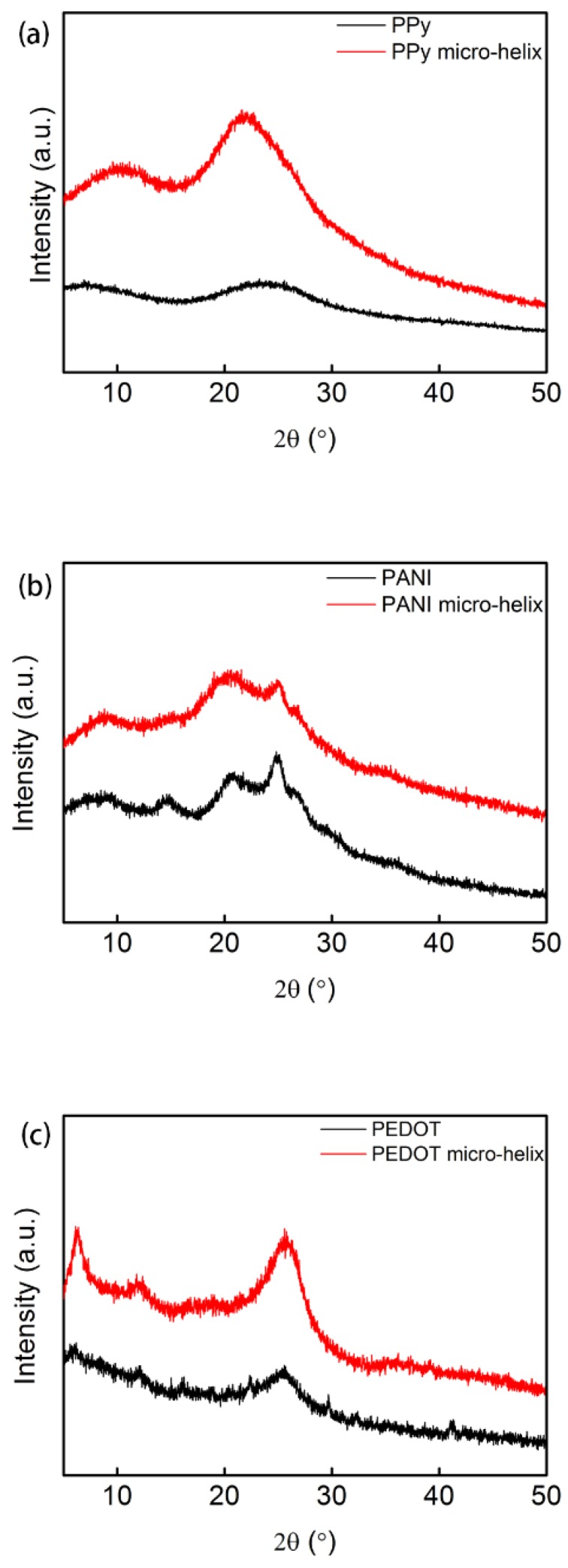
The X-ray diffraction (XRD) pattern of PPy and the PPy micro-helix (**a**); PANI and the PANI micro-helix (**b**); and PEDOT and the PEDOT micro-helix (**c**).

The crystal structures of PPy, the PPy micro-helix, PANI, the PANI micro-helix, PEDOT, and the PEDOT micro-helix were characterized by XRD analysis. Figure 5a shows the XRD pattern of PPy and the PPy micro-helix. The broad peaks in the region of 15–30° for both PPy and the PPy micro-helix are mainly due to the scattering from PPy chains at the interplanar spacing and indicate a typical form of amorphous polymer [88]. The peaks that shift from 23.2° in PPy to 21.6° in the PPy micro-helix suggest the interaction between the *Spirulina* template surface and PPy coating [89]. In Figure 5b, the peaks at 2θ = 14.8° and 25.0° in both PANI and the PANI micro-helix are caused by periodical perpendicularity and parallelism in the polymer chain, respectively. Additionally, the peak at 20.5° can be attributed to the alternating distance of the polymer chain layers for both PANI and the PANI micro-helix [90,91]. The broadened peaks at 2θ = 14.8° and 25.0° in the PANI micro-helix can be attributed to the intermolecular interaction between PANI and the *Spirulina* surface [92]. In Figure 5c, both PEDOT and the PEDOT helix have wide diffraction peaks in the region of 2θ = 22–28°, which suggest their amorphous forms [93,94]. The peak at 2θ = 25.4° in PEDOT shifted to 2θ = 25.6° in the PEDOT helix, which suggests the increase of the doping level [95,96].

### 3.5. Electrical and Electrochemical Properties

Four-point probes were used to determine the sheet resistance of the conducting polymer micro-helices without any extra disposition. The sheet resistances of the PPy micro-helix, the PANI micro-helix, and the PEDOT micro-helix are measured as 10.2 ± 0.60, 17.7 ± 2.22, and 42.2 ± 3.15 Ω/sq, respectively. The sheet resistance of the PPy micro-helix is close to that of the reported PPy/MWCNT/cotton (6.0 ± 0.4 Ω/sq) [97]. The sheet resistance of the PANI micro-helix is at the same level as that of the reported pristine PANI (about 5 Ω/sq) [98,99]. The sheet resistance of the PEDOT micro-helix is much better than that of the PEDOT/PET films (190 Ω/sq) [100].

For a single electrode, its specific capacitance, *C*_S_ (F/g) can be calculated from CV curves:

(1) $$\;C_{s}=\frac{\int IdV}{vm\Delta U}\;$$

where Δ*U* is the potential window, *ν* is the scan rate, *m* is the mass of the active material electrode, *I* is the current, and *V* is the voltage.

**Figure 6:**
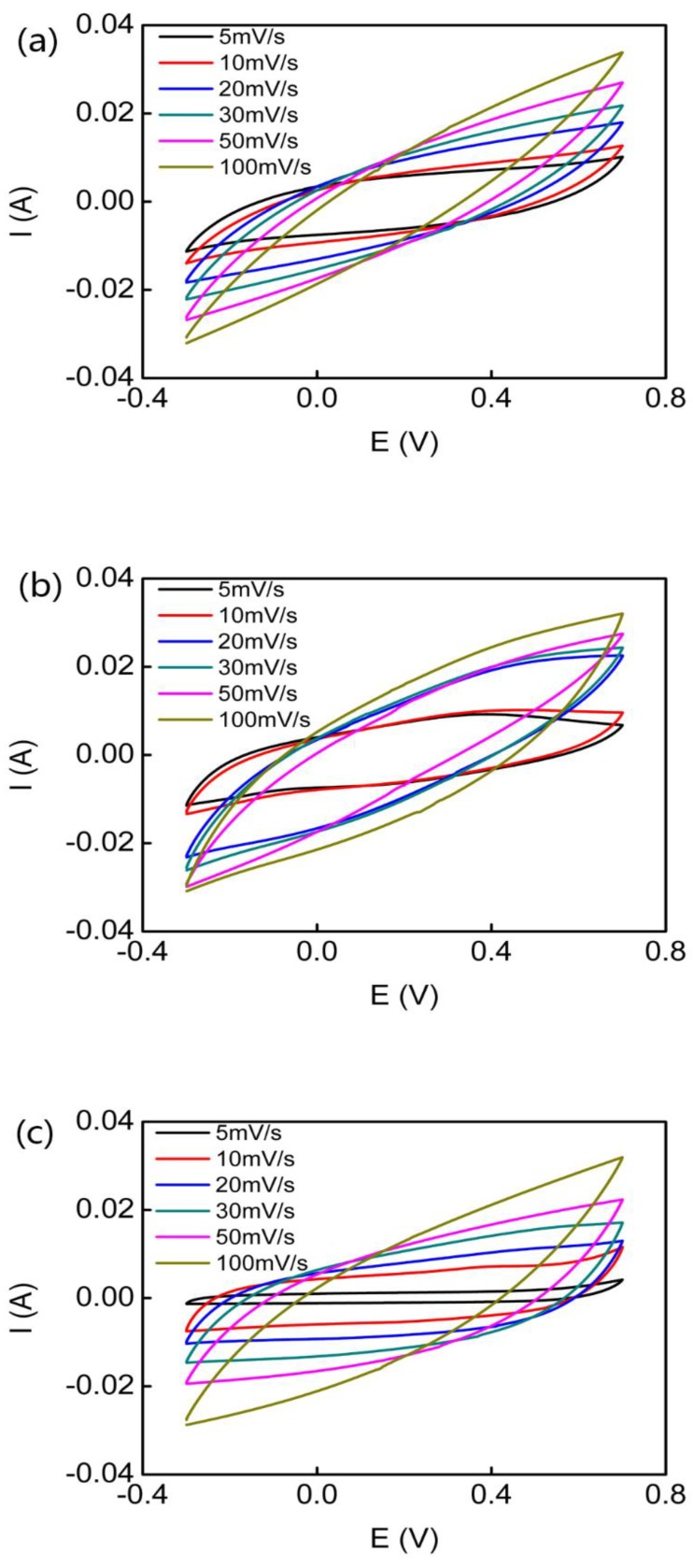
The cyclic voltammograms of the PPy micro-helix (**a**); the PANI micro-helix (**b**); and the PEDOT micro-helix (**c**) with different scan rates from 5 mV/s to 100 mV/s.

**Figure 7:**
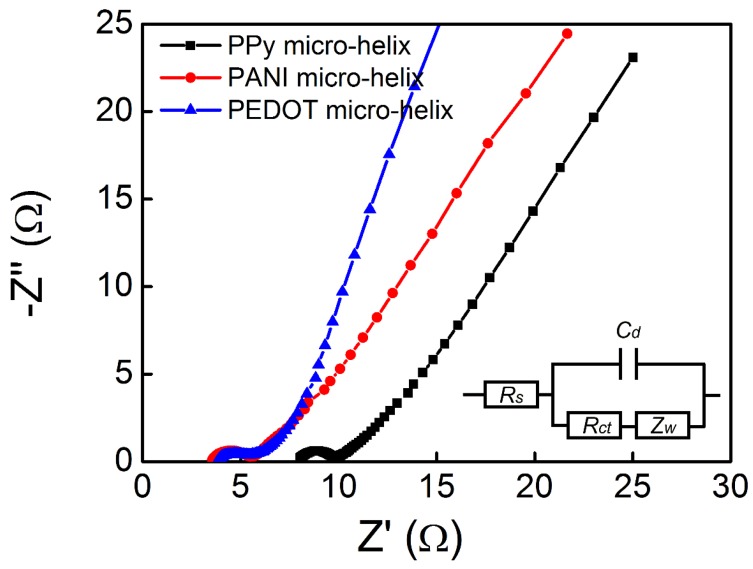
The Nyquist plots for the PPy micro-helix, the PANI micro-helix, and the PEDOT micro-helix in the frequency range of 10^5^ Hz to 0.01 Hz at an open circuit potential with an alternating current (AC) perturbation of 10 mV; inset: the electrical equivalent circuit for PPy, PANI, and the PEDOT micro-helix.

In Figure 6, the CV curves of the PPy micro-helix, the PANI micro-helix, and the PEDOT micro-helix with a series of scan rates exhibit a double-layer capacitance characteristic. In Figure 6a, the highest specific capacitance of the PPy micro-helix was found to be 234 F/g, which is almost the same as those of the reported PPy-coated cotton (268 F/g) and PPy-coated viscose rayon (244 F/g), and higher than those of the reported PMAS/PPy-coated Actitex carbon fiber (152.0 F/g) and PPy-coated acrylonitrile butadiene rubber (125.8 F/g) with the same scan rate [101,102,103]. In Figure 6b, the highest specific capacitance of the PANI micro-helix was found to be 238 F/g, which is slightly higher than that of the reported PMAS/PANI-coated Actitex carbon fiber (212 F/g) [102,104]. In Figure 6c, with the scan rate of 10 mV/s, the PEDOT micro-helix showed the highest specific capacitance of 106.4 F/g, which is higher than that of the reported CNTs/PEDOT composite (85/15, *w*/*w*) (70 F/g) [105]. EIS measurements were also carried out to study the characteristics of charge transfer in conducting polymers. An electrical equivalent circuit model for the conducting polymer micro-helix which is composed of electrolyte resistance (*R*_s_), double-layer capacitance (*C*_d_), charge transfer resistance (*R*_ct_), and Warburg impedance (*Z*_W_) was introduced to fit the EIS result, as shown in Figure 7 inset [97,106]. In the high-frequency region, the well-defined semicircles which represent charge transfer-dominated regimes and whose diameters represent the charge transfer resistance (*R*_ct_) are observed for all the micro-helices. The *R*_ct_ values of the PPy micro-helix, the PANI micro-helix, and the PEDOT micro-helix-modified electrodes are calculated as 1.666, 1.68, and 1.565 Ω, respectively, which is in the same level as those of the reported PPy/graphene electrode film (0.15–1.73 Ω) [107] and better than those of the reported PANI/MnWO_4_ composite (22 Ω) [108] and PEDOT-coated Au microelectrode arrays (749 Ω) [109]. This result suggests that the micro-sized *Spirulina* bio-template can effectively enlarge the specific surface of the conducting polymer, which facilitates counterion exchange on the electrode/electrolyte interface. The *R_s_* value of the PANI micro-helix and the PEDOT micro-helix are extracted as 3.737 and 4.108 Ω, respectively, which is much lower than that (8.201 Ω) of the PPy micro-helix, suggesting that the PANI micro-helix and the PEDOT micro-helix have low internal resistances for ion transfer in Na_2_SO_4_ aqueous solution.

## 4. Conclusions

In this study, we proposed a new strategy to fabricate conducting polymers in micro-helical form by using naturally-occurring *Spirulina* as a helical bio-template. In comparison with the conventional techniques for manufacturing micro-helices, *Spirulina*-templated conducting polymer micro-helices can be easily mass produced with an extremely low cost. The PPy micro-helix, the PANI micro-helix, and the PEDOT micro-helix were successfully synthesized in fine helical form. The strong interactions between the conducting polymers and the surface groups of *Spirulina* can be inferred from the multiple molecular structure analysis. The sheet resistances of the PPy micro-helix, the PANI micro-helix, and the PEDOT micro-helix are measured as 10.2 ± 0.60, 17.7 ± 2.22, and 42.2 ± 3.15 Ω/sq, and the optimized specific capacitances of the PPy micro-helix, the PANI micro-helix, and the PEDOT micro-helix were found to be 234, 238, and 106.4 F/g with scan rates of 5, 5, and 10 mV/s, respectively, which are comparable to other conducting polymer-coated micro-fiber materials. Moreover, this approach can be applied to produce other kinds of conducting polymer micro-helices and to further combine with functional nanomaterials with customized physical and chemical properties for making biomedical and microelectronic devices.
